# Neutrophil gelatinase-associated lipocalin as predictor of acute kidney injury requiring renal replacement therapy: A systematic review and meta-analysis

**DOI:** 10.3389/fmed.2022.859318

**Published:** 2022-09-21

**Authors:** Chunhua Xu, Shan Lin, Longyi Mao, Zesong Li

**Affiliations:** ^1^Guangdong Provincial Key Laboratory of Systems Biology and Synthetic Biology for Urogenital Tumors, Shenzhen Key Laboratory of Genitourinary Tumor, Department of Urology, The First Affiliated Hospital of Shenzhen University, Shenzhen Second People’s Hospital (Shenzhen Institute of Translational Medicine), Shenzhen, Guangdong, China; ^2^Guangdong Key Laboratory for Biomedical Measurements and Ultrasound Imaging, Shenzhen University Health Science Center, School of Biomedical Engineering, Shenzhen, Guangdong, China; ^3^Shulan International Medical College, Zhejiang Shuren University, Hangzhou, China; ^4^Department of Central Laboratory, Shenzhen Hospital, Beijing University of Chinese Medicine, Shenzhen, Guangdong, China

**Keywords:** neutrophil gelatinase-associated lipocalin, acute kidney injury, renal replacement therapy, predictive value, systematic review and meta-analysis

## Abstract

**Background:**

Patients with severe acute kidney injury (AKI) may require renal replacement therapy (RRT), such as hemodialysis and peritoneal dialysis. Neutrophil gelatinase-associated lipocalin (NGAL) is a sensitive indicator for early diagnosis and recognition of AKI; however, its predictive value of AKI-associated need for RRT needs further evaluation.

**Methods:**

Following the Preferred Reporting Items for Systematic Reviews and Meta-Analysis guidelines, relevant articles were systematically searched and selected from seven databases. The random effects model was applied to evaluate the predictive performance of NGAL for AKI requiring RRT. The Newcastle–Ottawa Scale (NOS) was used to assess the quality of each included study.

**Results:**

A total of 18 studies including 1,787 patients with AKI and having an average NOS score of 7.67 were included in the meta-analysis. For plasma/serum NGAL, the pooled sensitivity and specificity with corresponding 95% confidence interval (CI) were 0.75 (95% CI: 0.68–0.81) and 0.76 (95% CI: 0.70–0.81), respectively. The pooled positive likelihood ratio (PLR) was 2.9 (95% CI: 2.1–4.1), and the pooled negative likelihood ratio (NLR) was 0.34 (95% CI: 0.25–0.46). Subsequently, the pooled diagnostic odds ratio (DOR) was 9 (95% CI: 5–16) using a random effects model, and the area under the curve (AUC) of summary receiver operating characteristic to summarize predictive accuracy was 0.82 (95% CI: 0.79–0.85). For urine NGAL, the pooled sensitivity, specificity, PLR, NLR, DOR, and AUC values were 0.78 (95% CI: 0.61–0.90), 0.77 (95% CI: 0.65–0.85), 3.4 (95% CI: 2.4–4.8), 0.28 (95% CI: 0.15–0.52), 12 (95% CI: 6–24), and 0.84 (95% CI: 0.80–0.87), respectively.

**Conclusion:**

Plasma/serum and urine NGAL levels performed comparably well in predicting AKI requiring RRT. Our findings suggested that NGAL is an effective predictive biomarker for the AKI-associated need for RRT. Nevertheless, more pieces of high-quality evidence and future trials with larger sample sizes are needed for further improvement of patient outcomes.

**Systematic review registration:**

[https://www.crd.york.ac.uk/prospero/display_record.php?ID=CRD42022346595], identifier [CRD42022346595].

## Introduction

Acute kidney injury (AKI) has been emerged as a crucial public health issue, which affects millions of patients worldwide and is a common complication in patients hospitalized in the intensive care unit (ICU), and AKI is independently associated with significant morbidity and mortality (1–3). Non-AKI patients usually have better clinical outcomes than patients with AKI (4–6). The management of AKI usually involves several conservative interventions, such as avoidance of nephrotoxins and prompt resuscitation of circulation (4, 7). However, patients with AKI with severe metabolic disorders, such as acidosis, hyperkalemia, uremia, and fluid disorders, whose kidney function does not recover after certain interventions have to undergo renal replacement therapy (RRT) with a possibility of eventual kidney transplantation (5, 8). Despite considerable research on RRT, it is still unclear if and when RRT should be commenced to improve the outcome of patients with AKI (5, 9). Although early initiation of RRT may reduce the mortality of patients with AKI (10), it may cause a higher risk of treatment-related complications, such as bloodstream infections (11). More importantly, few studies have specifically evaluated the value of various biomarkers to predict AKI that may persist or worsen and progress to a certain stage, resulting in a necessary reception of RRT (12, 13). Therefore, a new biomarker that serves as an early predictor of AKI and an indicator of the need to undergo RRT may play a critical role in improving the prognosis of AKI.

Neutrophil gelatinase-associated lipocalin (NGAL), a secretory protein with a molecular weight of 25 kDa, belongs to the lipocalin superfamily and is released from injured tubular epithelial cells in response to various insults (5, 14). NGAL has already been acknowledged by nephrologists as one of the most promising biomarkers of upcoming AKI. Recently, plasma/serum and urine NGAL levels have been investigated as biomarkers for early prognostication of AKI (9). It is well known that AKI and chronic kidney disease (CKD) are interconnected syndromes as AKI may exacerbate CKD progression and CKD increases the risk of AKI. Serum and urinary NGAL levels were significantly higher in patients with CKD than in the normal population and were negatively correlated with the glomerular filtration rate (GFR). Although NGAL cutoff values and kinetics are significantly altered in patients with CKD, NGAL is considered not only a better indicator of a GFR decline than serum creatinine (sCr) but also a potent marker of the degree of kidney injury. According to the multivariate Cox proportional risk regression model, serum and urinary NGAL levels were independent predictors of the risk of CKD progression (15). Furthermore, several studies have reported that an increase in NGAL levels can be detected well before an increase in plasma creatinine (pCr) levels, which highlights the sensitivity of the former as a biomarker for diagnosing AKI (11, 16). For instance, pCr did not increase until 24–72 h postoperatively in patients undergoing elective cardiac surgery; however, increases in urine and plasma NGAL levels were identified as soon as 2 h postoperatively, with areas under receiver operating characteristic curve of 0.99 and 0.91, respectively (16). However, because of the lack of corresponding statistical data for early prediction of AKI requiring RRT (17–19), it remains controversial whether NGAL is a predictive biomarker of AKI requiring RRT. Therefore, the potential of NGAL for early prediction of AKI-associated RRT remains to be established.

To illuminate this issue, a systematic review and meta-analysis was conducted to evaluate the ability of the available physiological and molecular biomarkers for predicting the initiation of RRT in patients with AKI. This systematic review and meta-analysis was performed to explore the predictive evidence of AKI requiring RRT.

## Methods

### Data sources and searches

We performed a systematic search of the following databases: PubMed, Embase, the Web of Science, Cochrane Library (in English), Chinese National Knowledge Infrastructure,^1^ and Wanfang Data (in Chinese).^2^ The search duration was from inception to November 2021. The following search terms were used: [“Biomarkers” (MeSH) OR biomarker OR marker OR neutrophil gelatinase associated lipocalin OR NGAL OR neutrophil gelatinase-associated lipocalin] and (AKI OR acute kidney injury OR acute kidney failure OR acute renal failure), and (RRT OR renal replacement therapy). Abstracts with a complete section “Results” were included in this study. There were no language restrictions. This meta-analysis was conducted and reported according to the Preferred Reporting Items for Systematic Reviews and Meta-Analysis (PRISMA) statement issued in 2009 (Checklist file) (20, 21).

### Study selection

All citations were reviewed, and the literature was retrieved by titles or abstracts, and subsequently, full texts were reviewed by two investigators (CX and SL) to determine the study eligibility. Any disagreements regarding study eligibility were resolved by consulting another investigator (ZL). Studies meeting the following inclusion criteria were included: (1) studies with participants aged ≥ 18 years; (2) studies using plasma/serum and/or urine NGAL for prediction of patients with AKI who might need RRT; (3) studies including AKI and non-AKI patients with sepsis who underwent RRT; (4) observational studies; and (5) studies with enough information to calculate true-positive, false-positive, false-negative, and true-negative values of NGAL as a predictor of AKI requiring RRT (contains AUC, sensitivity, and specificity values) or studies with these values provided. Studies were excluded if they met the following exclusion criteria: (1) studies with only animal or *in vitro* experiments; (2) studies lacking the information about predictive accuracy in control or experimental groups; (3) review articles, commentaries, poster presentations, letters, supplementary issues, and editorials; (4) studies with duplicate data or insufficient information; and (5) studies on individuals with prior kidney transplant, end-stage kidney disease, or prior RRT.

### Data extraction and quality assessment

A total of two investigators (CX and SL) independently extracted the data from each trial, and any disagreements between them were resolved by consulting a third investigator (ZL) and reaching a consensus. Data on the following variables from each article were documented and recalculated: first author, year of publication, study location, population type, gender, total sample size, AKI definition, number of patients with AKI, number of patients undergoing RRT, age, NGAL assay results, sample type, AUC (95% CI), and NGAL cutoff, sensitivity, and specificity. Absolute data of true-positive (TP), false-positive (FP), true-negative (TN), and false-negative (FN) rates or equivalent data were calculated or extracted.

The two investigators (CX and SL) independently assessed the methodological quality of the studies using the Quality Assessment of Diagnostic Accuracy Studies (QUADAS) tool (22). This tool is based on four key domains: index test, reference standard, patient selection, and flow and timing. Each domain is evaluated in the aspect of “risk of bias,” and the first 3 domains are evaluated in the aspect of concern regarding applicability. The included studies collected response using “yes,” “no,” or “unclear” items. The responses of “yes” are considered positive responses for analysis herein.

The Newcastle–Ottawa Scale (NOS) was used to assess the quality of each included study (23). Based on several aspects of the study, such as comparability (maximum points, 2), outcomes (maximum points, 3), and selection (maximum points, 4), the quality of the study was judged using a “star” scoring system of NOS. The scores range from 0 (for worst) to 9 (for best). A study with a score no less than 7 was considered a high-quality study.

### Data synthesis and analysis

We used STATA version 12.0 (Stata Corp, College Station, TX) to perform all statistical analyses, which included TP, TN, FP, and FN rates for each test in every study, to assess the sensitivity, specificity, positive likelihood ratio (PLR), negative likelihood ratio (NLR), and diagnostic odds ratio (DOR) for each included study. Statistically significant heterogeneity was represented by *P* < 0.05 for *Q* statistic, and *I*^2^ > 50% was considered to indicate substantial heterogeneity (24). The degree of heterogeneity between multiple studies was measured using the *I*^2^ index, and *I*^2^ values of <25, 25–50, and >50% indicated modest, moderate, and substantial heterogeneity, respectively. A random effects model was chosen if *I*^2^ was greater than 50% (25). Any departure from the Hardy–Weinberg equilibrium (HWE) in the control group of each study was assessed using the χ^2^ test, and significant deviations were represented by *P* < 0.05 (26).

Forest plots of accuracy indices were constructed, and a summary receiver operating characteristic (SROC) curve was constructed on the basis of TP and FP rates to describe the relationship between test sensitivity and specificity. NGAL has been defined as a useful risk predictor when the AUC ≥ 0.70, and the predictive performance for the prediction of AKI in RRT by NGAL was measured by calculating the AUC as an overall summary index (27).

Furthermore, subgroup analyses were performed according to the biological material, definition of AKI, geographic location, NGAL assay method, and AKI causes. Finally, Begg’s and Egger’s measures were assessed and calculated using Begg’s funnel plots to detect publication biases (28, 29). A statistically significant difference was represented by *P* < 0.05 in the test results for the overall effect.

## Results

### Literature search

**FIGURE 1:**
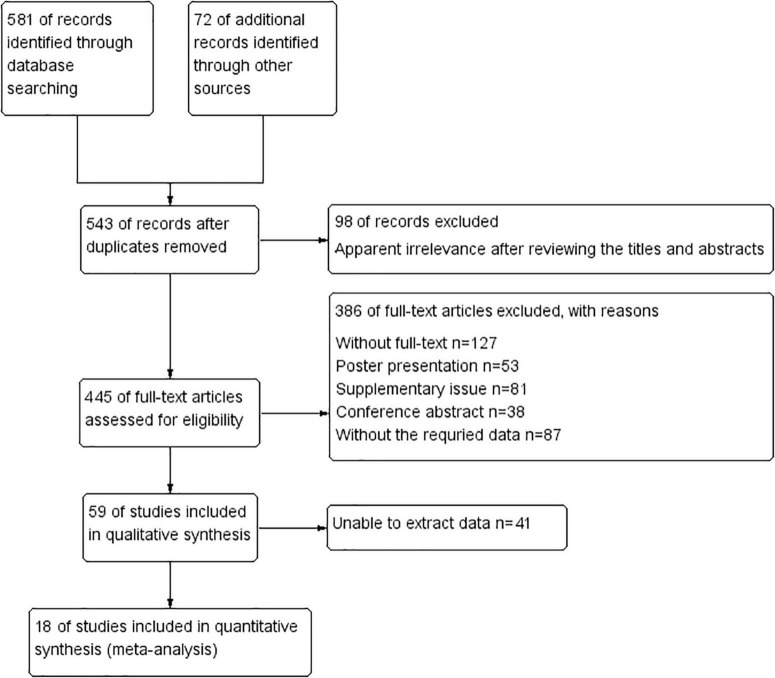
Selection process of the included studies in the meta-analysis.

During the literature search, we initially identified 653 potentially relevant studies, and 543 studies remained after removing the duplicates found in electronic databases. Subsequently, 98 articles were identified as irrelevant after reviewing titles and abstracts and excluded. Then, 445 full-text articles were assessed for eligibility, and 386 of these articles were excluded as they did not meet the requirements of data extraction. After screening the full texts of the remaining articles, 41 studies were excluded as they did not meet our eligibility criteria. Finally, 18 articles that met the inclusion criteria were included in this meta-analysis. These studies encompassed a total of 5,441 participants who were included in the meta-analysis for prediction of AKI requiring RRT. The selection process of the included studies is shown in Figure 1.

### Characteristics and quality of the included studies

All 18 selected articles were written in English. To analyze the quality of the included studies, the main characteristics were extracted, as presented in Table 1. The included studies were geographically diverse: 10 studies were conducted in Europe (14, 30–38), two in America (39, 40), one in Australia (41), and the remaining five studies in Asia (42–46). The 18 observational studies involved a total of 5,441 patients from 12 countries. All studies were single-center trials published between 2006 and 2021. Overall, 1,787 patients developed AKI and 637 patients received RRT. The use of plasma/serum NGAL and urine NGAL was almost equal among the studies. The definitions of AKI varied among individual studies. A total of 13 studies used the traditional method to define AKI, and the remaining five studies used a non-traditional method. Most studies evaluated the NGAL level in the plasma and urine, rather than in the serum. Commercial enzyme-linked immune sorbent assay (ELISA) was frequently used for NGAL measurements. Among the 18 studies, nine used ELISA and nine used other methods to measure the NGAL level. The performance of NGAL for predicting AKI requiring RRT is summarized in Table 2.

**TABLE 1:** Main characteristics of the studies included in the meta-analysis.

References	Location	Sample size (male)	AKI definition	Cause	Source	AKI (*n*)	RRT (*n*)	NGAL assay	NOS
Albeladi and Algethamy (42)	Saudi Arabia	75 (38)	NR	ICU	Urinary	21	17	NR	8
Chen et al. (43)	China	110 (NR)	KDIGO	Sepsis	Serum/urinary	110	78	NR	7
Chun et al. (44)	Korea	76 (66)	NR	Burn	Plasma	32	20	NR	8
Constantin et al. (30)	France	88 (NR)	RIFLE	ICU	Plasma	36	7	NR	7
Cruz et al. (31)	Italy	301 (207)	RIFLE	ICU	Plasma	133	15	NR	8
Cruz et al. (32)	Italy	933 (NR)	NR	Critically ill	Plasma	284	40	ELISA	7
Gaipov (37)	Turkey	60 (42)	KDIGO	Heart surgery	Urinary	40	7	ELISA	8
Hanson et al. (41)	Australia	163 (136)	NR	Malaria	Urinary	84	43	ELISA	8
Hjortrup et al. (33)	Denmark	222 (126)	NR	Sepsis	Plasma/urinary	91	29	NR	8
Kaufmann et al. (35)	Germany	255 (184)	KDIGO	Sepsis	Plasma	33	33	ELISA	8
Linko et al. (34)	Finland	369 (243)	RIFLE	ICU	Plasma	47	47	NR	8
Lukasz et al. (38)	Germany	39 (28)	AKIN	Uremia	Serum	31	24	ELISA	7
Maisel et al. (39)	United States	927 (575)	KDIGO	Heart failure	Plasma	72	11	NR	8
Nisula et al. (14)	Finland	1042 (673)	KDIGO	Critically ill	Urinary	379	83	ELISA	8
Park et al. (45)	Korea	169 (96)	KDIGO	Sepsis	Serum	114	114	NR	7
Tiranathanagul et al. (46)	Thailand	47 (31)	AKIN	Critically ill	Plasma/urinary	47	18	ELISA	7
Tornblom et al. (36)	Finland	484 (310)	KDIGO	ICU	Urinary	217	46	ELISA	8
Wagener et al. (40)	United States	81 (53)	AKIN	ICU	Urinary	16	5	ELISA	8

**TABLE 2:** Predictive value of NGAL on AKI requiring RRT in individual studies.

Study	AUC	95% CI	Cut-off value	Sensitivity	Specificity	Number of patients			
						TP	FP	FN	TN
Albeladi and Algethamy (42)	35.000	1.274–961.305	200 ng/mL	1.00	0.56	17	2	0	2
Chen et al. (43)	29.400	8.950–96.581	403 ng/mL	0.81	0.89	63	4	15	28
Chen et al. (43)	35.000	10.489–116.793	695 ng/mL	0.83	0.88	65	4	13	28
Chun et al. (44)	3.250	0.733–14.402	253 ng/mL	0.63	0.67	13	4	8	8
Constantin et al. (30)	15.750	1.630–152.179	303 ng/mL	0.90	0.72	6	8	1	21
Cruz et al. (31)	12.207	2.627–56.724	150 ng/mL	0.87	0.65	13	41	2	77
Cruz et al., (32)	3.107	1.485–6.501	NR	0.72	0.54	29	112	11	132
Gaipov (37)	20.172	1.064–382.451	6 ng/mL	0.94	0.58	7	14	0	19
Hanson et al. (41)	4.511	1.798–11.316	510 ng/mL	0.65	0.70	28	12	15	29
Hjortrup et al. (33)	4.040	1.573–10.376	641 ng/mL	0.69	0.64	20	22	9	40
Hjortrup et al. (33)	2.786	1.084–7.156	1832 ng/mL	0.46	0.77	13	14	16	48
Kaufmann et al. (35)	0.91	0.82–0.99	10.26 U/g	0.90	0.89	30	24	3	198
Linko et al. (34)	6.044	3.118–11.716	304 ng/mL	0.68	0.74	32	84	15	238
Lukasz et al. (38)	30.000	2.794–322.090	330 ng/mL	0.83	0.80	20	1	4	6
Maisel et al. (39)	18.375	3.504–96.363	125 ng/dL	0.80	0.80	9	12	2	49
Nisula et al. (14)	17.866	9.442–33.804	449 ng/mL	0.83	0.79	69	64	14	232
Park et al. (45)	2.405	1.209–4.783	576.5 ng/mL	0.61	0.58	70	23	44	32
Tiranathanagul et al. (46)	22.533	4.648–109.247	960 ng/mL	0.72	0.90	13	3	5	26
Tiranathanagul et al. (46)	10.833	2.383–49.242	2600 ng/mL	0.55	0.91	10	3	8	26
Tornblom et al. (36)	0.769	0.729–0.806	1000 ng/mL	0.53	0.92	24	14	22	157
Wagener et al. (40)	3.333	0.276–40.287	470 ng/mL	0.81	0.48	4	6	1	5

### Assessment of methodological quality and publication bias

**FIGURE 2:**
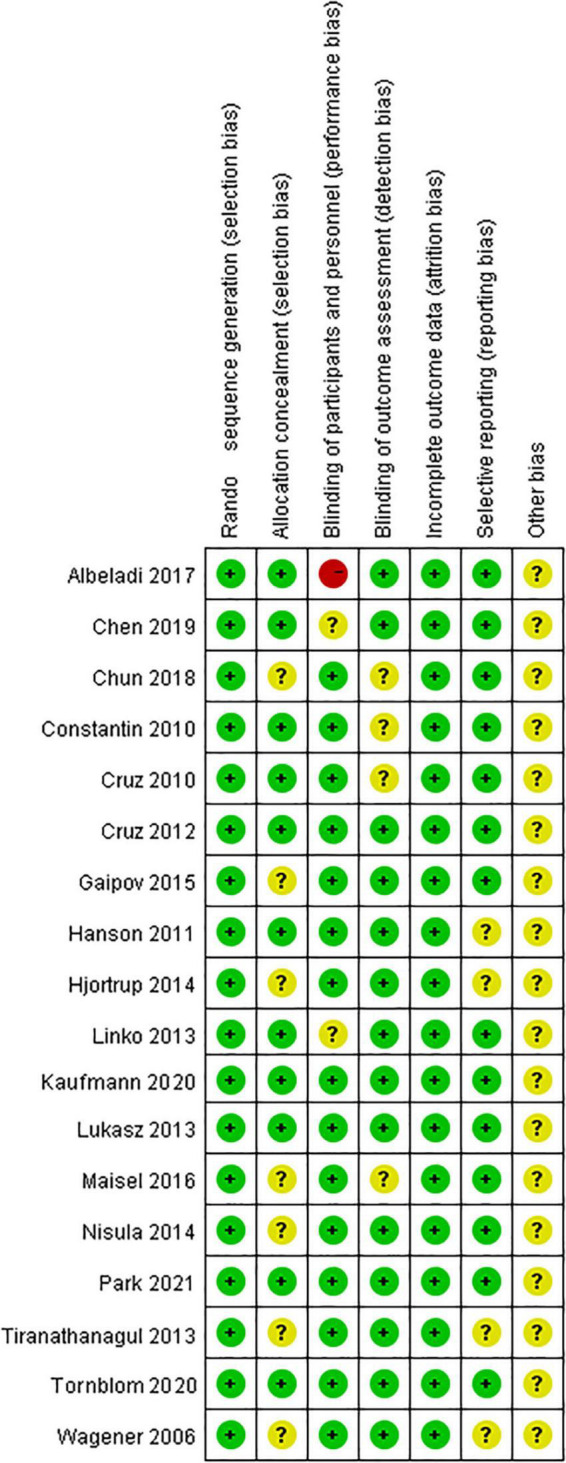
Quality assessment of included eligible studies using QUADAS-2.

All studies were clearly defined with eligibility criteria and reasons for patient exclusion. The quality of each included study was assessed using the QUADAS tool, with all of them having high QUADAS scores (≥10). The overall quality of included trials was moderate. The results of the QUADAS-2 evaluation are shown in Figure 2.

**FIGURE 3:**
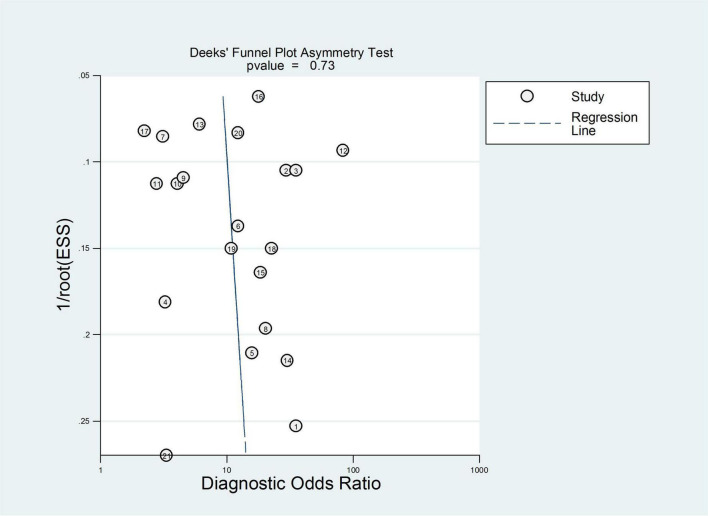
Deek’s funnel plot assessment of potential publication bias.

Subsequently, publication bias assessment was conducted using a funnel plot. The results of the funnel plot are shown in Figure 3, which indicated no significant threshold effect and no significant asymmetry, suggesting that there was no evident publication bias in the present meta-analysis. Therefore, it is unlikely that unpublished studies would substantially alter our findings.

### Neutrophil gelatinase-associated lipocalin for prediction of acute kidney injury requiring renal replacement therapy

**FIGURE 4:**
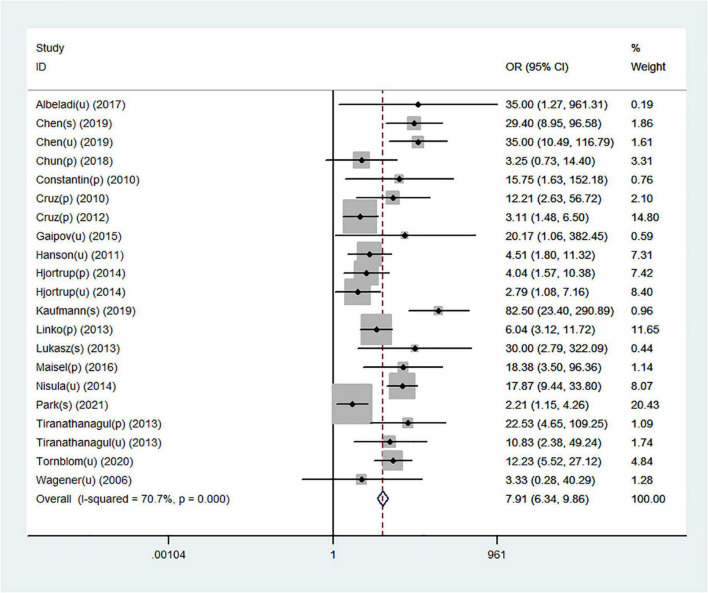
Forest plot for the predictive value of NGAL for AKI requiring RRT from plasma/serum and urine samples.

**FIGURE 5:**
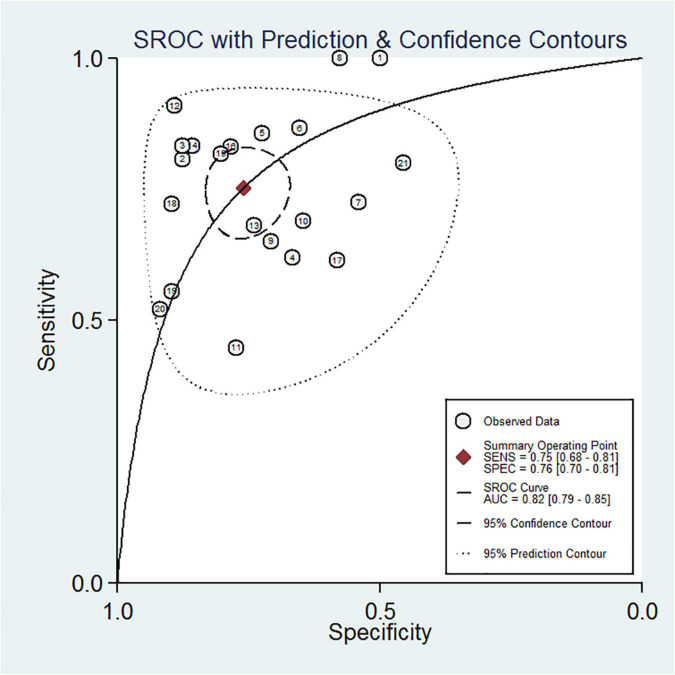
Summary receiver operating characteristic (SROC) curve of plasma/serum and urine NGAL for AKI requiring RRT.

Table 2 shows a total of 21 sets of data extracted from 18 eligible studies, including TP/FP/FN/TN value, sensitivity, specificity, positive predictive value, negative predictive value (NPV), AUC, various optimal cutoff values for different sample types of NGAL, the NGAL assay method, and the definition of AKI. In the 18 studies, we investigated the predictive value of plasma NGAL as a biomarker of AKI requiring RRT in 1787/5441 patients who developed AKI. The pooled results of these studies are summarized in Table 2. Taken together, the predictive value of NGAL for AKI requiring RRT from plasma/serum and urine samples is shown in Figure 4. For summary performance estimates, the pooled sensitivity and specificity with corresponding 95% CI were 0.75 (95% CI: 0.68–0.81) and 0.76 (95% CI: 0.70–0.81), respectively. The pooled PLR was 2.9 (95% CI: 2.4–4.1), and the pooled NLR was 0.34 (95% CI: 0.25–0.46). The pooled DOR was 9 (95% CI: 5–16) using a random effects model (Figure 4). Moreover, the AUC for SROC to summarize predictive accuracy was 0.82 (95% CI: 0.79–0.85; Figure 5). Even though the result of SROC for AKI requiring RRT was worse than that for AKI, its clinical application was still of great value.

### Subgroup analysis

Subgroup analysis of this meta-analysis was performed, and the results are shown in Table 3. Based on the comparisons of DOR and AUC, the predictive value of NGAL for AKI requiring RRT showed some variability. For subgroup analysis of the biological material, the DOR and AUC of the urine NGAL level were significantly higher than those of the plasma/serum NGAL level in the prediction of AKI requiring RRT (DOR, 12; AUC, 0.84 vs. DOR, 9; AUC, 0.82). This showed that urine NGAL performed better for RRT prediction than plasma/serum NGAL. For subgroup analysis of geographic location, the value of the NGAL level to predict AKI requiring RRT in oriental was substantially higher than that in occidental (DOR, 14; AUC, 0.85 vs. DOR, 9; AUC, 0.81). Subsequently, for subgroup analysis of the NGAL assay method, we investigated the predictive accuracies of NGAL with and without ELISA. The findings revealed that the former had a higher predictive value for AKI requiring RRT (DOR, 14; AUC, 0.86 vs. DOR, 7; AUC, 0.79). For subgroup analysis of the definition of AKI, the traditional definition of AKI was associated with better DOR and AUC values (DOR, 13; AUC, 0.85) than the non-traditional definition of AKI (DOR, 4; AUC, 0.70). Moreover, subgroup analysis for different causes of AKI was conducted, and the results showed that AKI caused by sepsis/heart failure had the best predictive ability with optimal DOR and AUC (DOR, 10; AUC, 0.83), and AKI caused by ICU showed a similar predictive ability as AKI caused by critical illness (DOR, 9; AUC, 0.82 VS DOR, 9; AUC, 0.80). Based on the establishment of subgroup analyses of the 21 datasets from the 18 studies wherein multivariable analyses were provided, it would be reasonable to believe NGAL as an independent predictor of AKI requiring RRT.

**TABLE 3:** Subgroup analysis on the basis of different standards.

Studies	Number		Sensitivity	Specificity	PLR	NLR	DOR	AUC-ROC
Biological material	12	Plasma/serum	0.75 (0.68–0.81)	0.76 (0.70–0.81)	2.9 (2.1–4.1)	0.34 (0.25–0.46)	9 (5–16)	0.82 (0.79–0.85)
	9	Urine	0.78 (0.61–0.90)	0.77 (0.65–0.85)	3.4 (2.4–4.8)	0.28 (0.15–0.52)	12 (6–24)	0.84 (0.80–0.87)
Definition of AKI	15	Traditional	0.78 (0.70–0.84)	0.79 (0.72–0.85)	3.7 (2.7–5.1)	0.28 (0.21–0.39)	13 (8–22)	0.85 (0.82–0.88)
	6	Non-traditional	0.69 (0.54–0.81)	0.63 (0.51–0.73)	1.9 (1.5–2.3)	0.49 (0.35–0.69)	4 (2–6)	0.70 (0.66–0.74)
Geographic location	14	Occidental	0.75 (0.66–0.83)	0.74 (0.67–0.81)	2.9 (2.2–3.9)	0.33 (0.24–0.46)	9 (5–15)	0.81 (0.78–0.85)
	7	Oriental	0.75 (0.67–0.82)	0.82 (0.70–0.89)	4.1 (2.3–7.3)	0.30 (0.20–0.45)	14 (5–35)	0.85 (0.81–0.87)
NGAL assay method	10	ELISA	0.78 (0.67–0.87)	0.80 (0.70–0.87)	3.9 (2.6–5.7)	0.27 (0.18–0.41)	14 (8–26)	0.86 (0.83–0.89)
	11	Others	0.73 (0.65–0.81)	0.73 (0.65–0.79)	2.7 (2.0–3.7)	0.37 (0.26–0.52)	7 (4–14)	0.79 (0.75–0.83)
Causes	6	ICU	0.80 (0.63–0.90)	0.70 (0.52–0.83)	2.6 (1.7–3.9)	0.29 (0.17–0.49)	9 (5–16)	0.82 (0.78–0.85)
	8	Sepsis/heart failure	0.75 (0.64–0.84)	0.77 (0.68–0.85)	3.3 (2.1–5.2)	0.32 (0.20–0.51)	10 (4–25)	0.83 (0.80–0.86)
	7	Critically ill	0.73 (0.64–0.80)	0.77 (0.65–0.85)	3.1 (2.0–4.9)	0.36 (0.26–0.49)	9 (4–18)	0.80 (0.76–0.83)

### Heterogeneity analysis

**FIGURE 6:**
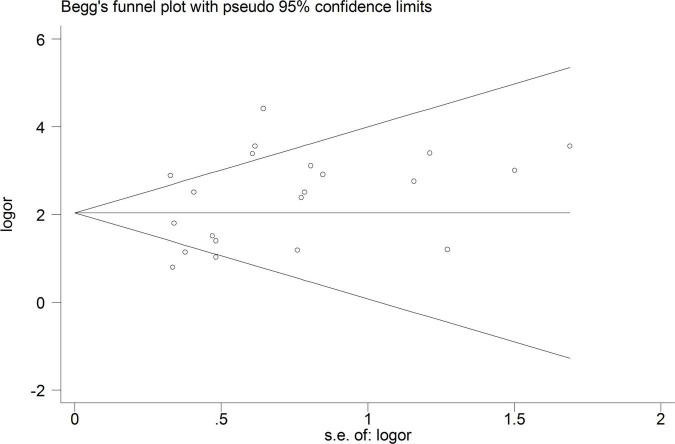
Begg’s funnel plot for testing publication bias.

A total of 18 heterogeneous studies containing 21 datasets of plasma/serum NGAL and urine NGAL for the prediction of AKI requiring RRT were obtained. Overall, heterogeneity analyses were performed, and the SROC was constructed; the points in the plots did not show a “shoulder arm” pattern, which suggested no presence of the threshold effect. Subsequently, Begg’s funnel plot and Egger’s test were used to check for publication bias, and the funnel plots are shown in Figure 6. The results indicated a low probability of publication bias.

To explore other possible reasons for heterogeneity, meta-regression and subgroup analyses were performed. The main sources of heterogeneity could be the geographic location (occidental or oriental) and definition of AKI (traditional or non-traditional). However, the specimen type (urine or plasma/serum) and the NGAL assay (ELISA or others) may not be the sources of heterogeneity for NGAL.

## Discussion

In the present systematic review and meta-analysis, we assessed the predictive accuracy of NGAL in 1,787 patients with AKI requiring RRT. The results of this meta-analysis revealed NGAL as a valuable renal biomarker to predict the need for RRT with high sensitivity, specificity, and DOR in patients scheduled to undergo AKI. Furthermore, subgroup analyses indicated that plasma/serum and urine NGAL had comparable predictive values for AKI requiring RRT. Moreover, the definition of AKI and/or the geographic location of the patients affected the efficiency of the NGAL level for predicting AKI requiring RRT. Of note, although the predictive value of NGAL has been shown in AKI requiring RRT, it is hard to suggest NGAL as a promising biomarker that may guide clinical decision regarding the timing of initiation of RRT among patients with AKI due to the lack of established cutoff values of NGAL for the initiation of RRT. In addition, whether mild AKI or severe AKI requiring RRT will be developed is difficult to be predicted only by a certain increase in NGAL levels. As a matter of fact, clinical manifestations and variables measured on clinical laboratory platforms are the common factors that should be taken into consideration in clinical practice to decide the initiation of RRT, such as severely decreased renal function indicated by sharply increased serum creatinine or dramatically decreased GFR, acidosis, severe edema, hyperkalemia, and so on. Furthermore, NGAL levels not only reflects kidney injury but may also be influenced by systemic conditions such as sepsis or originating from non-kidney tissues. A increase in NGAL indicates the possibility of AKI and provides appropriate preparations for subsequent possible therapies.

Neutrophil gelatinase-associated lipocalin has been implicated in a variety of processes, including cell differentiation, proliferation, and survival in renal epithelial cells, where it helps maintain the tubular structure and limits apoptosis (16). In addition, exogenous NGAL has been shown to have a dramatic renoprotective effect in the mouse models of renal ischemia–reperfusion injury (47). Generally, NGAL is among the most extensively studied biological markers for early prediction of AKI in both urine and blood specimens. A systematic review was performed by Haase-Fielitz et al. wherein they included 58 studies collectively encompassing >16,500 patients and reported that both plasma and urine NGAL were predictive of AKI, with overall AUCs ranging from 0.79 to 0.87 in different clinical settings (48). The predictive accuracy of plasma and urine NGAL for the prediction of AKI was systematically reviewed, and the pooled results indicated that plasma and urine NGAL precisely predicted AKI with sepsis (AUC = 0.86 and 0.90, respectively) (49). In addition, NGAL is considered to play a vital role in early prediction of AKI as its level can rapidly increase after contrast medium exposure (27). However, it remains controversial whether NGAL is predictive of AKI requiring RRT because of the lack of pertinent statistical data in this regard, and it remains unclear when and whether RRT should be commenced to improve the outcome of patients with AKI on the basis of NGAL levels. Currently, the information on NGAL for the prediction of RRT in patients with AKI is extremely limited.

Several studies have evaluated the predictive value of NGAL for AKI of different etiologies that requires RRT, and a wide range of predictive values of NGAL levels for AKI has been reported in observational cohort studies. However, few studies considered patients after AKI administration for RRT (50). Since it is still unclear whether and when to commence RRT, standards for prediction of AKI requiring RRT are considered as a major limitation of biomarker studies (51). Recently, two trials employed a preset NGAL threshold as an inclusion criterion for RRT prediction and used NGAL to guide the early initiation of RRT (9, 52). Nevertheless, NGAL was found to detect patients with AKI in the ELAIN trial, whereas it was universally elevated in the STARRT-AKI pilot trial and showed weak discriminative value between patients requiring and not requiring RRT (9). The results of our study were consistent with those of previous studies, and we found NGAL to be a useful early predictor of AKI requiring RRT; our sensitivity analyses also revealed that the findings were robust. The association between NGAL and AKI requiring RRT is further highlighted by a sensitivity of 75% and a specificity of 76%. By contrast, several studies have reported sensitivities of 40%–60% and specificities of 40%–55%. The observed differences may be attributed to variations in the definitions of AKI, the etiology of AKI, the NGAL assay, and the geographic location of patients. To evaluate the predictive value of the NGAL level in various conditions, our subgroup analysis had included these parameters.

Recently, plasma and urine NGAL were reported to have relatively low predictive values for the requirement of RRT in ICU patients with severe sepsis and without CKD, and the AUCs were 0.73 (95% CI: 0.61–0.85; *P* = 0.64) and 0.68 (95% CI: 0.53–0.83; *P* = 0.64), respectively (33). In addition, in a study of 126 patients with sepsis, 23 of 58 patients with septic AKI received RRT (53). The results showed that the peak urine NGAL was higher in patients receiving hemodialysis than in those not receiving hemodialysis (median, 456 ng/mL vs. 341 ng/mL, *P* < 0.0001). The AUC of the peak urine NGAL for predicting the need for hemodialysis was 0.77 (95% CI: 0.64–0.83), with a cutoff value of 494 ng/mL; the sensitivity and specificity were 0.89 and 0.71, respectively. In addition, a study performed a subgroup analysis of AKI patients with community-acquired pneumonia who met the RIFLE-F criteria and showed that plasma NGAL was a poor predictor of the requirement of RRT (AUC, 0.62; 95% CI: 0.45–0.81) (54). Another study reported that the peak plasma NGAL showed fair discriminatory power for the prediction of AKI (AUC = 0.71) and need for RRT (AUC = 0.78); however, urine NGAL did not perform equally well for prediction of AKI (AUC = 0.70) and need for RRT (AUC = 0.70). Together, these findings are not comparable with our findings in terms of both plasma/serum NGAL and urine NGAL in the present meta-analysis for predicting AKI requiring RRT (55). Furthermore, a recent study conducted by Albert C. et al. also showed that urinary and plasma NGAL concentrations may identify patients at high risk for AKI and the associated need for dialysis therapy (56).

Currently, NGAL is the only biomarker that has been investigated in both plasma/serum and urine for its early predictive value of AKI and AKI requiring RRT. Therefore, herein, we assessed the predictive value of plasma/serum and urine NGAL and further examined the studies that conducted comparisons of both plasma/serum NGAL and urine NGAL for subgroup analysis. Regarding plasma/serum NGAL, many studies showed unsatisfactory results, and our subgroup analysis also showed that plasma/serum NGAL may have inferior performance to urine NGAL in prediction of AKI requiring RRT. Typically, plasma/serum NGAL is considered an indicator of systemic inflammation and not of renal injury. However, urine sample collection is non-invasive, and urine has reduced interfering proteins, thus making it an ideal fluid for kidney biomarker discovery. The present meta-analysis confirmed the superiority of urine NGAL for the prediction of AKI requiring RRT, suggesting urine NGAL levels should be quantified before plasma/serum NGAL levels. Conversely, it may be difficult to obtain urine samples from AKI patients with severe oliguria, which is common in cardiac surgery. Therefore, even though urine NGAL has a higher predictive value (DOR, 12; AUC, 0.84) than plasma/serum NGAL (DOR, 9; AUC, 0.82) for early predictive value of AKI requiring RRT, plasma/serum NGAL may be an alternative to urine NGAL in case urine is unobtainable. Subsequently, in the present study, subgroup analyses showed the definition of AKI as the main source of heterogeneity. Compared with the traditional definition of AKI (DOR, 13; AUC, 0.85), the non-traditional definition of AKI was found to be associated with an inferior predictive value of NGAL (DOR, 4; AUC, 0.70). To our knowledge, few studies have conducted a comparison of research-based assays. Therefore, prospective studies with geographic location or multiple RRT settings involving head-to-head comparisons may contribute to a more comprehensive evaluation of NGAL in prediction of AKI requiring RRT.

Nevertheless, this study has several limitations. First, the literature search strategy applied in this study was limited to open-access publications. In this case, the studies that may have met the inclusion criteria of this meta-analysis but were not published on open-access platforms were missed. Second, different cutoff values for NGAL were applied in the included studies. Furthermore, cutoff values were corrected by urine creatinine, and it was difficult to determine the optimized overall cutoff value for patients with AKI (57–59). Moreover, deeper investigations should be pursued incorporating a wide range of clinical settings of AKI. Third, the pooled ORs were calculated by the numbers of genotypes or alleles of controls and cases; however, no adjustment was performed for other confounding factors. Fourth, because of the limitation of statistical power, the results from subgroups analysis should be interpreted with caution. Finally, the included studies in the present meta-analysis had different match variables, and this may have affected the pooled results.

## Conclusion

The present systematic review and meta-analysis showed a significant association between NGAL and AKI requiring RRT. Therefore, NGAL could be considered a useful marker with a high predictive value of AKI requiring RRT. Despite these encouraging findings, in further studies, a larger sample of homogeneous patients should be used, and different NGAL assay methods need to be unbiased to clarify this issue. Furthermore, similar relevant studies incorporating long-term follow-up studies are needed to confirm the role played by NGAL in the progression and development of AKI.

## Data availability statement

The original contributions presented in this study are included in the article/supplementary material, further inquiries can be directed to the corresponding author.

## Author contributions

CX, SL, and ZL extracted the data and performed the initial analysis. CX and SL wrote the first draft, which has been carefully reviewed and edited by ZL. CX, SL, LM, and ZL performed further review and subsequent revisions. All authors have contributed to the conception and design of the study and agreed to the submission for publication.

## Footnotes

1
http://www.cnki.net/

2
http://www.wanfangdata.com.cn
